# Pesticide Nanoformulations Based on Sunlight-Activated Controlled Release of Abamectin

**DOI:** 10.1021/acsomega.3c08015

**Published:** 2024-02-20

**Authors:** Selin Oyku Gundogdu, Ozgur Saglam, Ali Arda Isikber, Huseyin Bozkurt, Hayriye Unal

**Affiliations:** † Faculty of Engineering and Natural Sciences, Sabanci University, Istanbul 34956, Turkey; ‡ SUNUM Nanotechnology Research Center, Sabanci University, Istanbul 34956, Turkey; § Faculty of Agriculture, Namık Kemal University, Tekirdağ 59030, Turkey; ∥ Agriculture Faculty, Plant Protection Department, Kahramanmaraş Sütçü Imam University, Kahramanmaraş 46100, Turkey

## Abstract

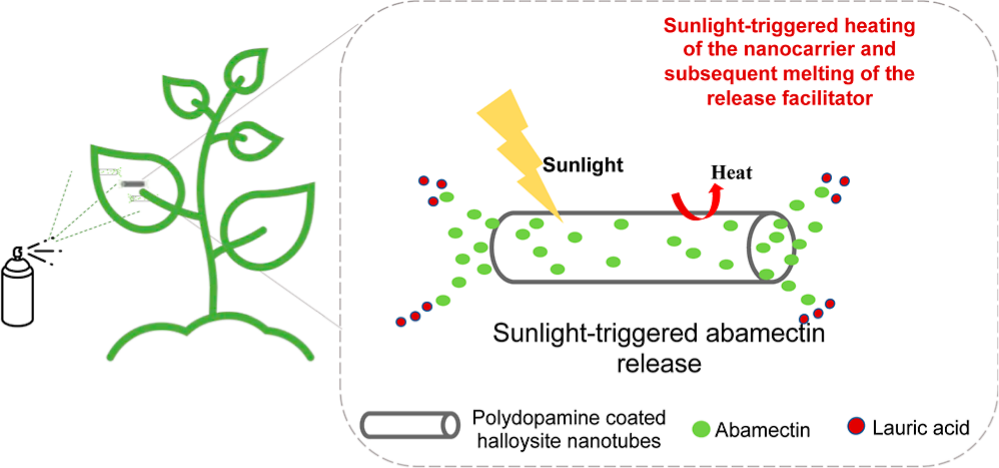

A controlled release system that enables the sunlight-triggered release of a model agrochemical, abamectin (abm), is presented. The release system consists of polydopamine functionalized halloysite nanotubes (HNT-PDA) utilized as photothermal nanocarriers to encapsulate 25 wt % abm and 37 wt % lauric acid (LA), a phase change material, that acts as a heat-activable gatekeeper stopping or facilitating the abm release. When exposed to sunlight for 20 min at 1 and 3 sun light density, the temperature of the photothermal nanocarriers reaches 51 and 122 °C, respectively, which triggers the melting of LA and the consequent release of abm from the nanocarriers. Abm was shown to be released gradually over a period of 10 days when nanohybrids were exposed to sunlight for 6 h per day and to remain stable and kill *Myzus persicae* (Sulzer) (Hemiptera: Aphididae), green peach aphids, at a mortality rate of over 70% for at least 10 days. Aqueous dispersions of the LA/abm@HNT-PDA nanohybrids were studied in terms of their potential as aqueous sprayable pesticide nanoformulations and presented over 30% suspensibility, 36 mg/cm^2^ foliar retention, strong rainwater resistance, and a 50% mortality rate for *M. persicae* at a concentration of 9 mg/mL. The proposed sunlight-activated controlled release system based on photothermal, LA-functionalized HNT-PDA nanocarriers holds great potential as controlled release pesticide nanoformulations.

## Introduction

Pesticides are essential for crop protection and enhancing agricultural productivity.^1^ However, their indiscriminate use can cause negative drawbacks to both human health and the environment.^2^ Conventional pesticide formulations may lead to adverse ecological and environmental problems due to the majority of the applied pesticides being lost to the environment and only less than 1% being effectively utilized at the intended target.^3^ As an alternative approach, the delivery of the pesticides can be accomplished by stimuli-responsive controlled release systems, which prolong the lifetime of the pesticide molecules and make them resistant to external factors such as rainwater leaching or photodegradation, thereby enhancing their effectiveness and enabling the use of lower dosages.^4−7^ Controlled pesticide release systems triggered by different external stimuli have been demonstrated in the literature.^8−10^ Xiang et al. designed a pH-responsive attapulgite-based hydrogel pesticide release system and characterized its release behavior in aqueous solutions at varying pH values.^11^ Kaziem et al. developed an enzyme-responsive insecticide delivery system based on surface-functionalized hollow mesoporous silica loaded with insecticide and capped with α-CD, which is designed to release the pesticide cargo upon hydrolysis triggered by the presence of α-amylase.^12^ Even though these designs demonstrate the triggered release of pesticides in novel ways, the fact that they require an external stimulus that might not be readily available in agricultural environments and are challenging to apply in real applications limits their implementation in agriculture. Controlled pesticide systems designed to be triggered by effortless, practical, and accessible stimuli are needed for the practical utilization of these systems. Sunlight stands out among the various external triggers due to its continuous availability, zero cost, and nontoxic nature and offers a powerful alternative as a trigger for agrochemical delivery systems. The most practical way to exploit sunlight as a controlled release trigger is to utilize photothermal materials that can convert sunlight to heat and design release systems that are triggered by temperature elevations. A limited number of studies have demonstrated light-driven controlled release systems where the pesticide is released by light via photothermal effects.^13,14^ However, a sunlight-triggered pesticide delivery system that is composed of natural, low-cost components, is easy to manufacture and apply in the field, and yet presents strong pesticide activity has not been reported.

Halloysite nanotubes (HNTs) with hollow tubular structures are naturally occurring inorganic clay nanoparticles, making them suitable for use as environmentally friendly nanocarriers.^15−18^ HNTs were commonly utilized as scavenging agents in food packaging applications,^19^ as drug delivery vehicles for cancer therapy,^20^ or as fillers for improving the mechanical properties of composites.^21^ Due to their open-ended and positively charged Al_2_O_3_ inner lumen and negatively charged SiO_2_ outer surface porous structure, HNTs have been mainly used as nanocarriers for the encapsulation of varied active substances such as pigments,^22^ essential oils,^16,23−25^ dyes,^26^ or drugs.^27,28^ HNTs have also been employed as carriers for pesticide delivery. Sustained pesticide release systems have been demonstrated, wherein HNTs are incorporated into polymeric matrices^29−31^ or emulsions,^32^ thereby slowing down the release of loaded pesticides. Additionally, a limited number of HNT-based pesticide release systems have also been reported, in which the loaded pesticide is released from functionalized HNTs triggered by external stimuli, such as pH^33^ and temperature.^34^ However, an HNT-based, sunlight-activated controlled release system that can be employed as effective, sprayable, and environmentally friendly pesticide nanoformulations has not been demonstrated.

In this work, we designed a novel HNT-based sunlight-triggered controlled pesticide release system that allows pesticides to be released only under sunlight and keeps the pesticide when no stimuli are present in the environment. The release system is composed of a photothermal nanocarrier loaded with pesticide molecules and a heat-activable release facilitator that helps release the pesticide upon sunlight-activation of the photothermal nanocarrier. While HNTs functionalized with polydopamine,^35^ a promising photothermal agent due to its robust NIR adsorption via excellent efficiency in light-to-heat conversion,^36^ have been utilized as the photothermal nanocarrier; lauric acid (LA), a temperature-sensitive phase change material was utilized as a heat-activable release facilitator. LA was expected to present a melting transition when the photothermal HNT-PDA nanocarriers are heated under sunlight irradiation and led to the release of the loaded pesticide. The uniqueness of this study, which distinguishes it from other studies reported in the literature, is that it is the first example not only of the use of photothermal agents with light-to-heat conversion properties in pesticide release allowing sunlight activation but also of photothermally triggered pesticide release from HNTs, which are natural, environmentally friendly, low-cost, and nontoxic carriers. We have previously demonstrated that a similar design allows sunlight-activated release of a volatile molecule, carvacrol.^37^ Here, we demonstrate the utilization of the HNT-based sunlight-triggered release system for the delivery of abamectin (abm), a nonvolatile, widely used commercial insecticide,^38^ and the use of this release system in the form of aqueous sprayable nanoformulations presenting strong foliar retention properties and strong insecticidal activity on the green peach aphid *Myzus persicae* (Sulzer).

## Experimental Section

### Chemicals

HNTs were supplied by ESAN Eczacıbaşı. Dopamine (3-hydroxytyramine hydrochloride) was purchased from Acros Organics Inc. (Geel, Belgium). Ultrapure Tris base (Tris(hydroxymethyl) aminomethane) was acquired from MP Biomedicals, LLC (Irvine CA, USA). abm was purchased from Sigma-Aldrich (US). Agrimec EC with 18 g/L abm was obtained from Syngenta (Turkey). LA was purchased from Merck (Darmstadt, Germany). Extra pure methanol (99.8%) was obtained from Tekkim Ltd. (Bursa, Turkey). Pure water was obtained using a Milli-Q Plus system. All chemicals were utilized without any further purification.

### HNT-PDA Nanocarriers

As previously reported, HNT-PDA nanocarriers were prepared via oxidative polymerization of the dopamine monomer in the presence of HNTs.^39^ In an ice bath, HNTs were dispersed in purified water (10 mg/mL) with ultrasonication (Qsonica, Q700) for 30 min at 40% amplitude with a 3 s pulse on and 2 s pulse off. Then, dopamine monomer was added to the dispersion at 8 mg/mL concentration, and the pH was brought to 8.5 with Tris. The mixture was stirred at 30 °C for 24 h. By centrifugation at 11,000 rpm for 10 min, HNT-PDAs were isolated from the reaction mixture. The obtained sample was rinsed six times with water to remove any residue of Tris-base and unreacted dopamine. The prepared HNT-PDAs were dried in a vacuum oven at 80 °C for 24 h.

### abm@HNT-PDA and LA/abm@HNT-PDA Nanohybrids

Based on a previously published method,^40,41^ HNT-PDAs were impregnated with abm and LA using a solvent-assisted impregnation approach followed by vacuum treatment to produce the abm@HNT-PDA and LA/abm@HNT-PDA nanohybrids. The HNT-PDAs were dried for 24 h at 100 °C in a vacuum oven before impregnation. A solution consisting of 0.4 g of abm and 40 mL of methanol was prepared, and 0.6 g of HNT-PDAs were added. The resulting mixture was subsequently subjected to bath sonication for 20 min. Additionally, 0.5 g of LA was added to the abm@HNT-PDA nanohybrid, and the mixture was sonicated for 5 min to give the LA/abm@HNT-PDA nanohybrid.

TGA [Shimadzu Corp. DTG-60H (TGA/DTA)] was utilized to determine the amounts of loaded abm and LA. TGA was run with a scan range of 30–1000 °C and a heating rate of 10 °C/min under a nitrogen flow. eqs 1 and 2 were used to determine the precise weight ratios of abm and LA in the LA/abm@HNT-PDA nanohybrids
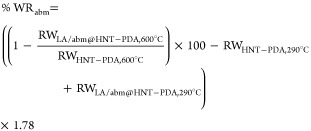
1
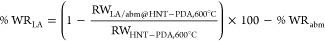
2where % WR_abm_ and % WR_LA_ denote the encapsulated abm and LA ratios in the nanohybrids, respectively, and RW_nanohybrid name,*x*°C_ denotes the remaining weight of the indicated nanohybrid at the given temperature (see details in Supporting Information Note 1).

Equation 3 was used to determine the weight ratio of abm in the abm@HNT-PDA nanohybrids
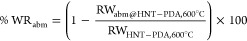
3where % WR_abm_ denotes the encapsulated abm ratio in the nanohybrid, and RW_nanohybrid name,*x*°C_ denotes the remaining weight of the indicated nanohybrid at the given temperature (see derivation of the equations in Supporting Information Note 1).

Differential scanning calorimetry (DSC) (Thermal Analysis MDSC TAQ2000) was utilized for investigating the thermal properties of the developed abm@HNT-PDA and LA/abm@HNT-PDA nanohybrids and neat abm. With a heating rate of 10 °C/min and a temperature range of 25–300 °C, the experiments were carried out in a nitrogen atmosphere.

Using a secondary electron detector with a 5 kV acceleration voltage, scanning electron microscopy (SEM) images of abm@HNT-PDA and LA/abm@HNT-PDA nanohybrids were acquired. Powder samples were sputter coated with Au/Pd.

Time–temperature profiles of the nanohybrids were constructed by recording temperatures using a FLIR E6xt thermal camera, while 0.5 g of abm@HNT-PDA and LA/abm@HNT-PDA powders were placed individually in Teflon holders under the solar simulator (Oriel LCS100) at 3 sun (300 mW/cm^2^) and 1 sun (100 mW/cm^2^) light densities. Three samples were tested for each time–temperature profile; mean and standard error values were reported.

Absorbance spectra were recorded with an Agilent Carry 5000 UV–vis–NIR spectrophotometer between the spectral range from 200 to 800 nm. abm@HNT-PDA (0.5 g) exposed to sunlight at 3 sun light density for 6 h and 0.5 g of abm@HNT-PDA stored in the dark were each mixed with 30 mL of methanol with the aim of releasing and dissolving abm in methanol. The abm@HNT-PDA and methanol mixtures were then centrifuged to precipitate the HNT-PDA nanocarriers, and the supernatant was then gathered for UV–vis examination. For the stability analysis of neat abm, 0.2 g of neat abm was dissolved in 30 mL of methanol, and the UV–vis spectrum was acquired before and after being exposed to sunlight at 3 sun light density for 6 h.

### abm Release Profiles of abm@HNT-PDA and LA/abm@HNT-PDA Nanohybrids

The release of abm from the nanohybrids in powder form was evaluated by TGA. For the release experiments, 0.4 g of abm@HNT-PDA and LA/abm@HNT-PDA nanohybrid powders were weighed precisely and placed on plates lined with a filter paper. Two sets of samples were prepared; one was incubated in the dark, and the other one was irradiated with sunlight at 3 sun for 6 h, followed by 18 h of dark incubation. All samples were wetted by adding 2 mL of deionized water at the end of 1 and 2 h incubation. The procedure was repeated for 10 days. Each day, three samples were taken from different parts at the end of a 6 h incubation, inserted into TGA crucibles, and analyzed. The abm content in the LA/abm@HNT-PDA and abm@HNT-PDA nanohybrids was calculated for each day based on eqs 1 and 2, respectively. Three measurements were taken for each sample, and the average encapsulated abm is determined. The % abm release was calculated with eq 4.
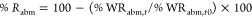
4where % *R*_abm_ denotes the percentage of released abm, % WR_abm,*t*0_ denotes the weight ratio of initially encapsulated abm in nanohybrids, and % WR_abm,*t*_ denotes the weight ratio of encapsulated abm in the nanohybrid at the specified time.

### Insecticidal Activity of Neat abm on *M. persicae*

abm solutions in acetone were prepared at 18 (recommended dose), 180, and 1800 g/mL. Petri dishes (6 cm diameter) containing agar were lined with eggplant leaves, sprayed with the prepared abm solutions using a spraying pump, and dried for 4 h. Green peach aphids were placed on the leaves and live/dead counts were conducted at the end of first, third, and fifth days. All tests were performed at 25 ± 2 °C and 70 ± 5% relative moisture.

### Insecticidal Activity of abm@HNT-PDA Powder on *M. persicae*

The green peach aphids (*M. persicae*) were maintained as a laboratory culture on eggplant leaves in an environmental chamber at 25 ± 3 °C temperature and 60–70% relative humidity under a photoperiod of 16 h light and 8 h dark. The nanohybrid (0.5 g) to be tested in the powder form was placed on filter paper. The nanohybrid samples were exposed to sunlight at 3 sun for 6 h. The samples were wetted with 2 mL of deionized water every hour during sunlight irradiation. Subsequently, the powders were removed from under the light source, and immediately 10 apterous adult aphids were placed on them using a sterile swab. After 15 min, the number of dead aphids was counted and the percentage of mortality was calculated. The same procedure was applied to samples of 0.5 g of LA/abm@HNT-PDA and 0.5 g of abm@HNT-PDA nanohybrids that were not exposed to light. The aphid tests were performed in triplicate.

### Suspensibility of the LA/abm@HNT-PDA Nanohybrids in Water

The suspensibility of LA/abm@HNT-PDA nanohybrids at 18, 9, and 1 mg/mL concentrations in deionized water was investigated. Prepared dispersions were subjected to bath sonication for 15 min and kept aside for 15 min; afterward, 500 μL of each were collected from the center using a micropipette and centrifuged to precipitate the nanohybrids. The precipitated nanohybrids were weighed after the supernatant was removed to determine the suspensibility ratios with eq 5.
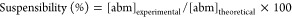
5

### Dispersion Analysis of Nanohybrids in Water

Aqueous dispersions of LA/abm@HNT-PDA were prepared at 4 mg/mL concentration and subjected to ultrasonication using a QSonica, Q700, Newtown, CT, USA, at 60% amplitude, with a 5 s pulse on and a 2 s pulse off for 30 min. Dynamic light scattering (DLS) measurements of aqueous dispersions were carried out using a Malvern Zetasizer Nano ZS (Malvern Instruments Ltd., U.K.) at 15, 30, 45 and 60 min to determine the size distribution and agglomeration state of nanohybrids.

### Determination of Leaching of abm into the Soil

Five grams of soil was mixed with 30 mL of 5 mg/mL aqueous LA/abm@HNT-PDA dispersion and incubated in the dark for 24 h. Following the incubation, the liquids were removed by vacuum filtration via a Buchner funnel, and the soil mixture was dried overnight at room temperature. The FTIR spectrum of the dried sample was obtained using a Thermo Scientific Nicolet Is10 FTIR spectrophotometer. As the control sample, 0.04 g of neat abm powder was mixed with 5 g of soil. The FTIR spectrum of the soil and abm mixture was obtained.

### Retention of the LA/abm@HNT-PDA Dispersion on the Leaves

A method reported in the literature was used for the calculation of leaf retention rates.^42^ Aqueous LA/abm@HNT-PDA nanohybrid dispersions, Agrimec, and abm dissolved in methanol were prepared at varying abm concentrations. Fifteen milliliters of sample in a beaker was placed on a sensitive balance. Leaves that had been cut into 2 × 2 cm squares, with a surface area of *S* = 4 cm^2^, were immersed into the liquid to be tested for its retention for 15 s and were removed and held up inside the weighing chamber until there were no droplets left on the surface. The decrease in the weight of the liquid (*W*) was recorded, and the retention was reported as 1000 × *W*/*S* (mg/cm^2^).

### Foliar Retention of the LA/abm@HNT-PDA Dispersion by Contact Angle Measurements

Aqueous dispersions of LA/abm@HNT-PDA were prepared at concentrations of 18, 9, 4.5, 2.25, 1.15, and 0.6 mg/mL abm and bath-sonicated for 15 min. The air pressure was set to 15–35 psi when the airbrush for spray coating (Magicbrush Airbrush Kit Ab-101a) was assembled in a chemical hood. The chamber of the airbrush was filled with the prepared LA/abm@HNT-PDA dispersion. The airbrush nozzle was held 10 cm in front of a set of eggplant leaves that had been cut into 2 × 2 cm squares positioned 30° from the surface axis, and each leaf sample was manually sprayed for 10 s with 10 mL of the dispersion. The spray-coated leaves were dried at room temperature.

The sessile-drop contact angle method was performed on both the pristine leaf samples and the leaf samples coated with spray-applied nanohybrids. This was accomplished using an optical tensiometer-equipped Theta Lite Contact Angle Measurement System. The optical tensiometer, which was equipped with a high-resolution digital camera, was used to measure the contact angles after 10 μL of pure water was carefully dropped on the surface at room temperature. Each sample had a minimum of three measurements made, and the average contact angle values were reported.

Contact angle measurements were also taken after sprayed leaf samples were washed with water to simulate rainfall after the spray application of LA/abm@HNT-PDA nanohybrid dispersion at a 9 mg/mL abm concentration. Each wash cycle involved spraying 1 mL of water with an airbrush at a spraying angle of 30°, resulting in 0.25 mL/cm^2^ sprayed water on the eggplant leaf. In between each wash cycle, leaf samples were dried at room temperature for 15 min. After the samples had dried, contact angle measurements were then taken from at least three spots of the samples.

### Determination of Insecticidal Activity

The insecticidal activity of the LA/abm@HNT-PDA dispersion against the green peach aphid was determined by spraying aqueous LA/abm@HNT-PDA nanohybrid dispersion, Agrimec EC diluted in cyclohexanol and abm dissolved in methanol, at abm concentrations ranging from 0.6 to 18 mg/mL, onto eggplant leaves cut into 2 cm × 2 cm squares. The airbrush nozzle was held 10 cm in front of a set of leaves that were positioned 30° from the surface axis, and the leaves were manually sprayed with formulations of tested pesticides for 10 s. The samples were exposed to sunlight at 3 sun for 1 h. Immediately following the sunlight irradiation, ten apterous adult aphids were placed on the leaves using a sterile swab. The dead aphids were counted after 15 min, and the mortality rate of the green peach aphid was calculated for each formulation dose. Similarly, another set of formulations sprayed on the leaf surface was prepared, and ten apterous adult aphids were placed on the leaves. The dead aphids were counted after 15 min, and the mortality rate of *M. persicae* was calculated for each formulation dose. The aphid tests were done in triplicate.

## Results and Discussion

**Figure 1:**
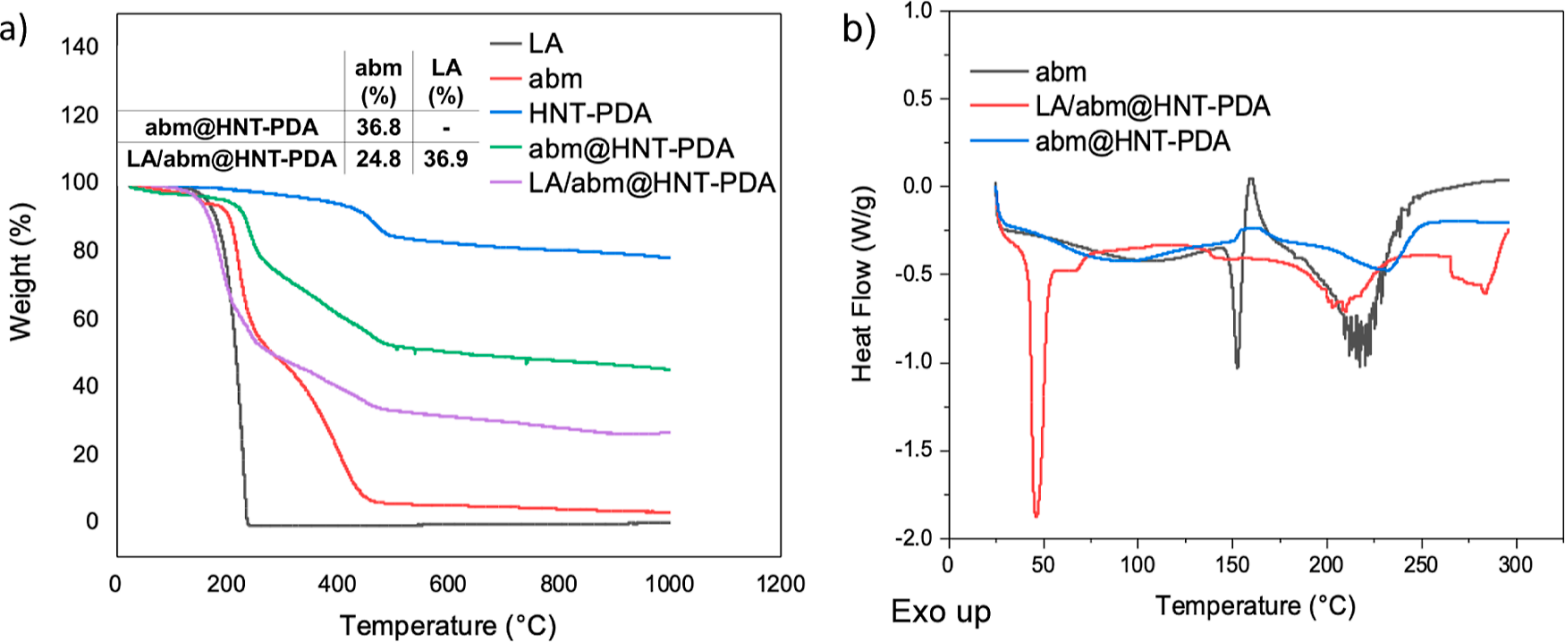
(a) TGA of HNT-PDA, LA, abm, abm@HNT-PDA, and LA/abm@HNT-PDA. (b) DSC of abm, abm@HNT-PDA, and LA/abm@HNT-PDA.

HNT-PDA nanohybrids, which were employed as photothermal carriers for the encapsulation of abm were prepared via the oxidative polymerization of dopamine on HNT carriers.^35^ HNT-PDA nanohybrids were loaded with abm via solvent-assisted impregnation.^37^ The resulting abm@HNT-PDA nanoparticles were further loaded with LA using the same method, resulting in LA/abm@HNT-PDA nanohybrids. TGA was employed to calculate the experimental loading ratio of abm and LA in the nanohybrids by using the relative weight loss ratios of neat and loaded abm and LA. Figure 1a demonstrates that the abm loading was 36.8% in abm@HNT-PDA nanohybrids, which were theoretically loaded with abm at 40 wt %. For the LA/abm@HNT-PDA nanohybrids, which theoretically contained 26.6 wt % abm and 33.3 wt % LA, the abm and LA loading ratios were 24.8 and 36.9%, respectively, demonstrating that both components were successfully loaded into the nanohybrids. The slight variations from the theoretical loading ratios might have been caused by the encapsulation of LA in the pores of the HNTs and some abm loss during solvent-assisted impregnation. DSC was further utilized to characterize the thermal properties of the LA/abm@HNT-PDA nanohybrids. Figure 1b shows that the nanohybrid presents a melting transition of LA around 50 °C, which was expected to facilitate the release of abm when the HNT-PDA nanocarriers are heated. Furthermore, the melting transition of abm at around 200 °C was shifted to a higher temperature. This finding indicates that some of the abm molecules entrapped in the HNT-PDA nanocarriers melted at higher temperatures when LA was present, confirming that the abm-loaded HNT-PDA nanocarriers were coated with LA. The SEM analysis of the abm@HNT-PDA and LA/abm@HNT-PDA nanohybrids also demonstrated that the LA functionalization resulted in an evenly distributed coating over the surface of the abm loaded HNT-PDA nanocarriers while the HNT-PDA nanocarriers retained their nanotubular structure (Figure S1).

**Figure 2:**
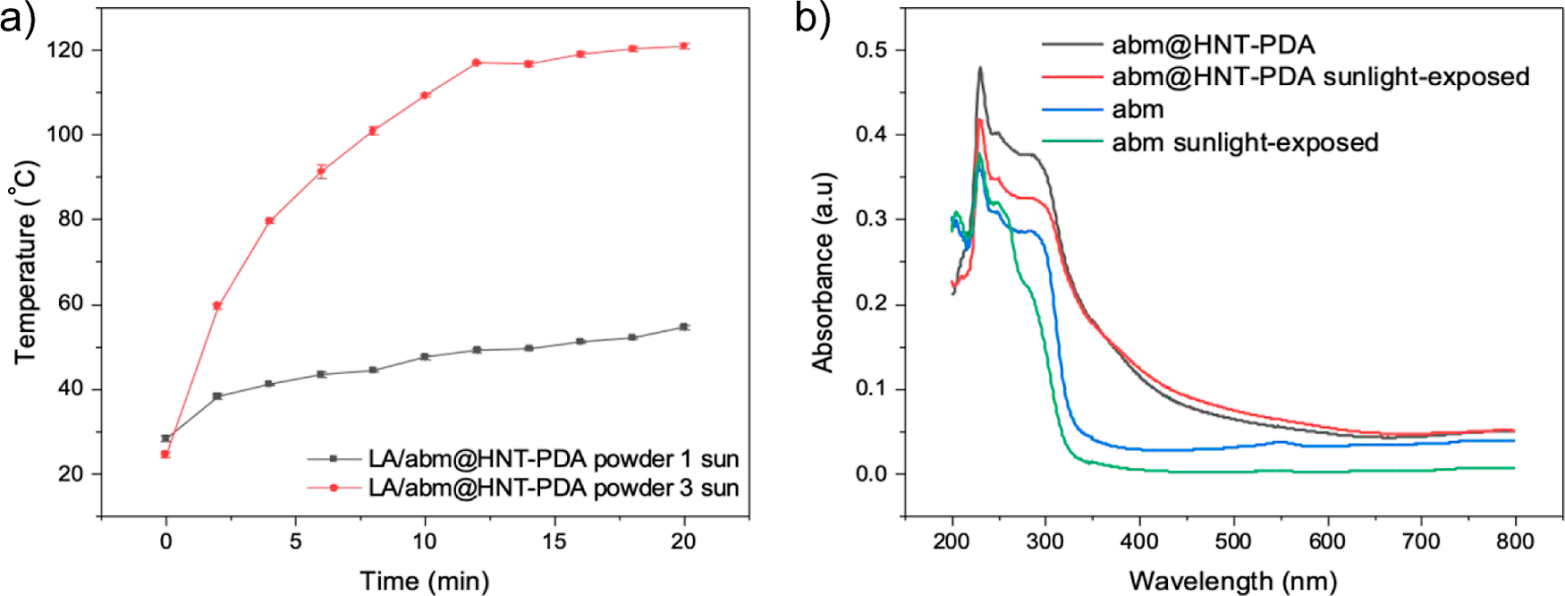
(a) Time–temperature profiles of LA/abm@HNT-PDA nanohybrids under irradiation at 1 sun and 3 sun light densities. (b) UV–vis absorption spectra of aqueous abm solution and abm encapsulated in HNT-PDAs before and after exposure to sunlight at 3 sun for 6 h.

The ability of LA/abm@HNT-PDA nanohybrids to undergo light-activated heating and achieve temperatures that may initiate the release mechanism upon exposure to sunlight was examined. By observing the temperature elevations upon irradiation from a solar simulator, the time–temperature profiles of nanohybrids were constructed (Figure 2a). When LA/abm@HNT-PDA nanohybrids were exposed to sunlight at a light density of 1 sun, they reached 50 °C. The temperatures were further elevated when the light density was raised to 3 sun, and the nanohybrids were heated to 120 °C. These findings demonstrated that the LA/abm@HNT-PDA nanohybrids can be heated up to desired temperatures in a controlled manner when exposed to sunlight at different light densities. Furthermore, it was demonstrated that the release system can be heated above the temperatures required for the melting transition of the LA release facilitator under sunlight irradiation.

abm is known to be prone to degradation when exposed directly to solar irradiation, which results in a short half-life and low utilization rate.^43^ Numerous kinds of encapsulation strategies have been designed in order to enable abm to retain its effectiveness for an extended period of time.^44−46^ Whether encapsulation in HNT-PDA nanocarriers enhanced the stability of abm against sunlight was studied by monitoring the changes in absorbance spectra of neat abm solution and abm encapsulated in HNT-PDAs before and after sunlight exposure. The absorbance spectra of encapsulated abm before and after sunlight were found to overlap (Figure 2b). However, a substantial drop in the characteristic absorbance peak of nonencapsulated abm between 200 and 300 nm was observed after sunlight exposure. This finding explicitly illustrates that the photostability of abm is preserved by its encapsulation with HNT-PDA.

**Figure 3:**
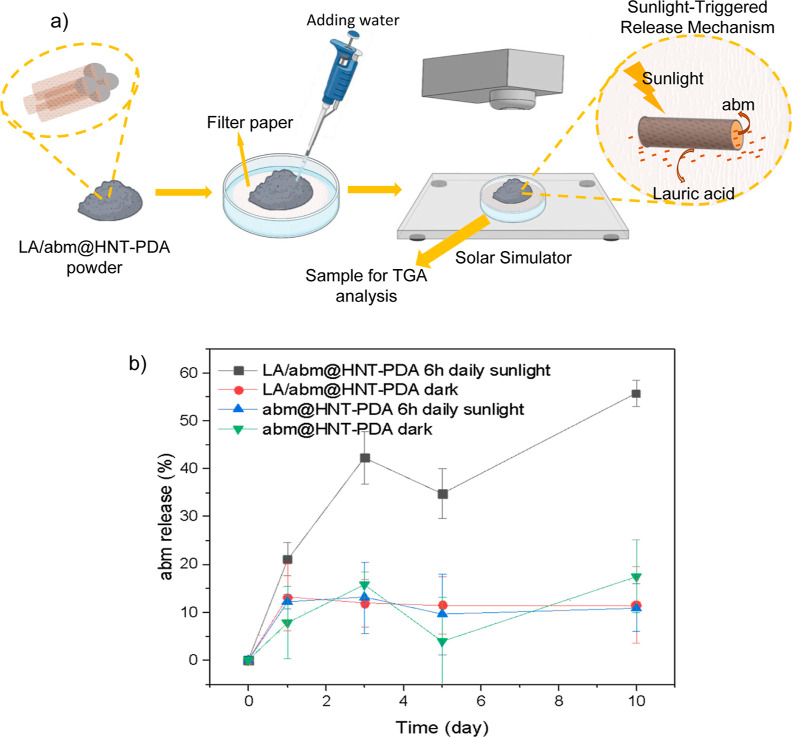
(a) Schematic representation of the experimental design to monitor the release behavior of abm from nanohybrids. (b) abm release from LA/abm@HNT-PDA and abm@HNT-PDA nanohybrids which were (i) irradiated with sunlight at 3 sun for 6 h each day and (ii) kept in the dark.

Figure 3a displays the experimental design for investigating the release behavior of sunlight-exposed LA/abm@HNT-PDA and abm@HNT-PDA samples. The nanohybrid samples were continuously wetted on filter paper simulating a moist plant surface that would retain the released abm and irradiated with sunlight for 6 h, followed by 18 h of dark incubation for 10 consecutive days. As controls, another set of each nanohybrid sample was kept in the dark without any sunlight activation for the same duration. Figure 3b demonstrates that the LA/abm@HNT-PDA nanohybrids released 55% of the encapsulated abm over the course of the 10 day period when they were irradiated with sunlight for 6 h each day. When they were not exposed to the sunlight, however, the abm release was not significant, confirming that the abm release was triggered with sunlight irradiation. Under the same conditions, abm@HNT-PDA nanohybrids, which were not functionalized with LA did not present a significant abm release when irradiated with sunlight for 6 h each day, demonstrating that LA acted as a release facilitator, which melts upon sunlight-activated heating of the HNT-PDA nanocarrier and eases the release of abm. The abm@HNT-PDA and LA/abm@HNT-PDA nanohybrids kept in the dark also exhibited almost no release at the end of the 10 day period, as predicted. At the end of the first 3 days, a burst release appeared in the samples exposed to sunlight. This was attributed to the release of abm absorbed on the outer surface of HNT-PDA nanocarriers as opposed to abm entrapped in the lumen, which was released more slowly. All of these findings support the idea that abm is being released in response to sunlight exposure and the phase transition of LA. The fact that the release efficiency was reduced in samples without LA confirmed the role of LA as a release facilitator in the proposed sunlight-triggered controlled release system.

**Figure 4:**
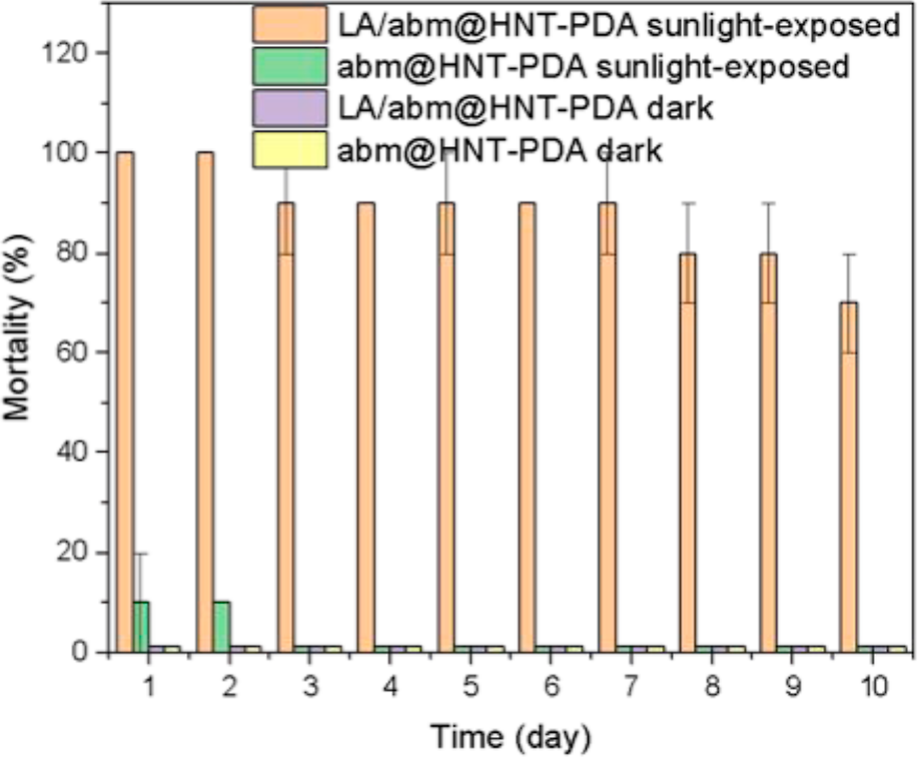
Mortality of *Myzus persicae* treated with LA/abm@HNT-PDA and abm@HNT-PDA nanohybrids, which were (i) exposed to 6 h sunlight at 3 sun each day and (ii) kept in the dark.

The effect of sunlight-triggered abm release from the LA/abm@HNT-PDA nanohybrids on the viability of *M. persicae* aphids was examined. First, the insecticidal activity of neat abamectin on *M. persicae* aphids was assessed by determining the mortality rates at different concentrations. The results confirmed a robust insecticidal activity, as shown in Figure S2. Over the course of a period of 10 days, the LA/abm@HNT-PDA nanohybrids were irradiated with sunlight for 6 h each day, at the end of which aphids were placed on the sunlight-irradiated powder samples. The viability of the aphids was recorded, and new aphids were placed on the powder after it was irradiated again the next day. The LA/abm@HNT-PDA nanohybrids presented 100% mortality on the first day when irradiated with sunlight and significantly retained their pesticide activity over a period of 10 days (Figure 4). Even after being exposed to sunlight for 10 days, the aphids treated with the nanohybrids presented 70% mortality, confirming that the LA/abm@HNT-PDA controlled release system presents a sunlight-activated release of abm and effective insecticidal activity over at least 10 days. Under the same conditions, the abm@HNT-PDA nanohybrids, which were not functionalized with LA did not present any pesticide activity, confirming that no significant release of abm occurred even under sunlight when LA was not present. After 10 days of incubation in the dark, neither the LA/abm@HNT-PDA nor the abm@HNT-PDA nanohybrid powders had an impact on the mortality of aphids. All these findings illustrated that LA functions effectively as a release facilitator in the abm release mechanism and that the controlled release mechanism only allows abm to be released from the nanohybrids when sunlight is available, which leads to mortality of the aphids. abm is not being released from the nanotubes in the absence of sunlight or in an environment where LA does not undergo phase transition, and consequently it does not present any insecticidal activity.

**Figure 5:**
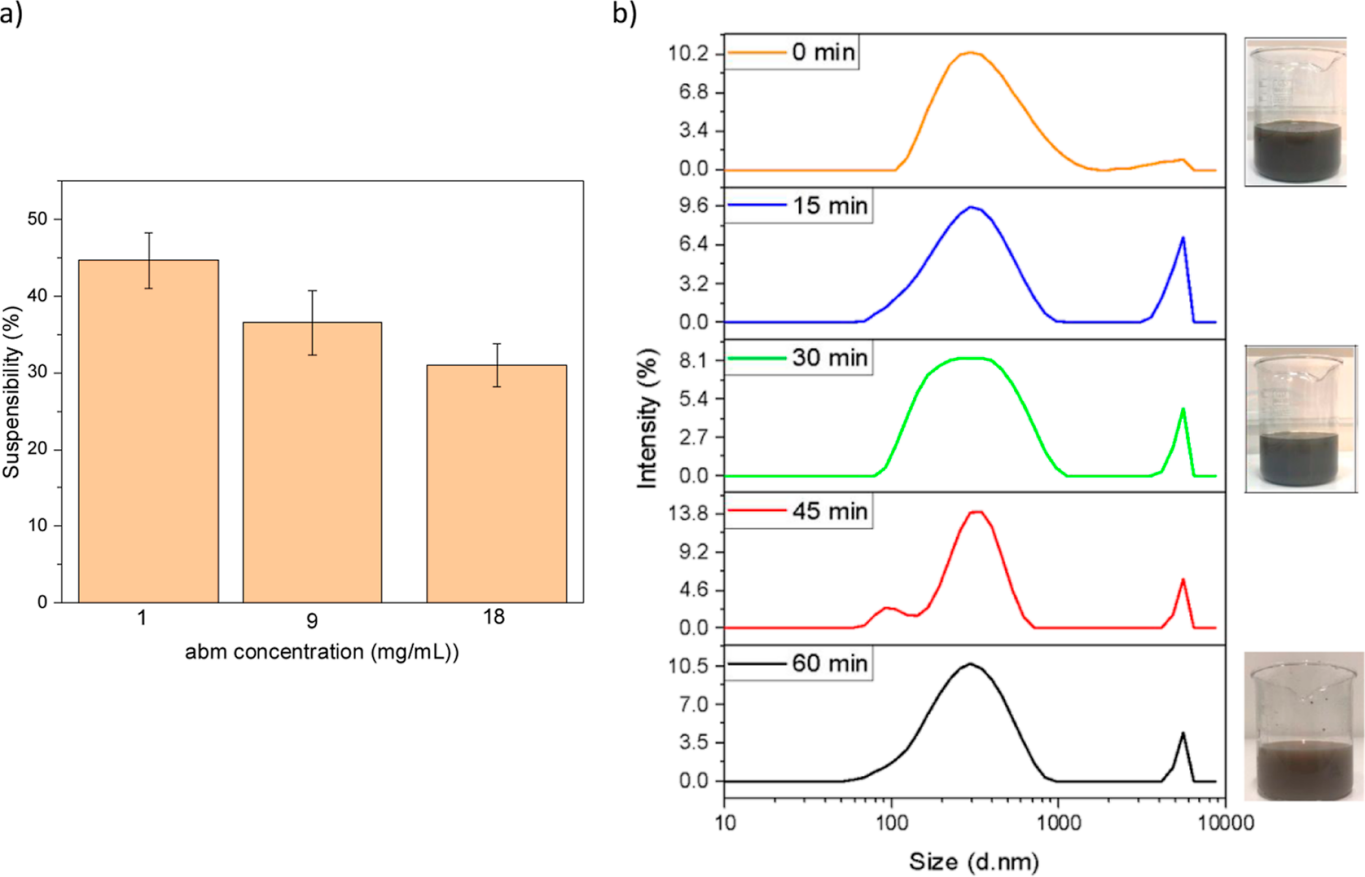
(a) Suspension test for LA/abm@HNT-PDA nanohybrids in water at different abm concentrations. (b) DLS analysis and photographs of the aqueous dispersion of LA/abm@HNT-PDA nanohybrids prepared at 4 mg/mL at different time periods.

To evaluate the potential of the sunlight-triggered release system in agricultural applications, their aqueous dispersions were studied as sprayable nanoformulations. LA/abm@HNT-PDA nanohybrids were dispersed in water at different abm concentrations and were examined using suspensibility analysis. As shown in Figure 5, the LA/abm@HNT-PDA nanohybrids were easily suspended in water with a suspensibility of above 30%. Apparently, the PDA functionalization of the HNT nanocarriers imparted hydrophilic character, which allowed the HNT-PDA nanocarriers to be suspended easily in water. As expected, the agglomeration of the HNT-PDA nanocarriers has increased at higher concentrations, and nanoformulations with lower concentrations presented a greater ability to be dispersed in water. The fact that the LA/abm@HNT-PDA can be easily suspended in water, without the need of any organic solvent, demonstrated their potential as environmentally friendly sprayable pesticide formulations.

The dispersion stability of the LA/abm@HNT-PDA nanohybrids was qualitatively evaluated using DLS analysis (Figure 5b). The size distributions of an aqueous dispersion of LA/abm@HNT-PDA nanohybrids at 4 mg/mL were analyzed at different time periods. While agglomeration of particles started to occur with an increase in time as seen by the new peak at higher hydrodynamic diameters, the size distribution of LA/abm@HNT-PDA dispersion did not significantly change over the period of 1 h. This result demonstrated that the LA/abm@HNT-PDA nanoparticles were mainly stable during the course of 1 h, which will allow spray-application of the developed sunlight-triggered release system in the field.

**Figure 6:**
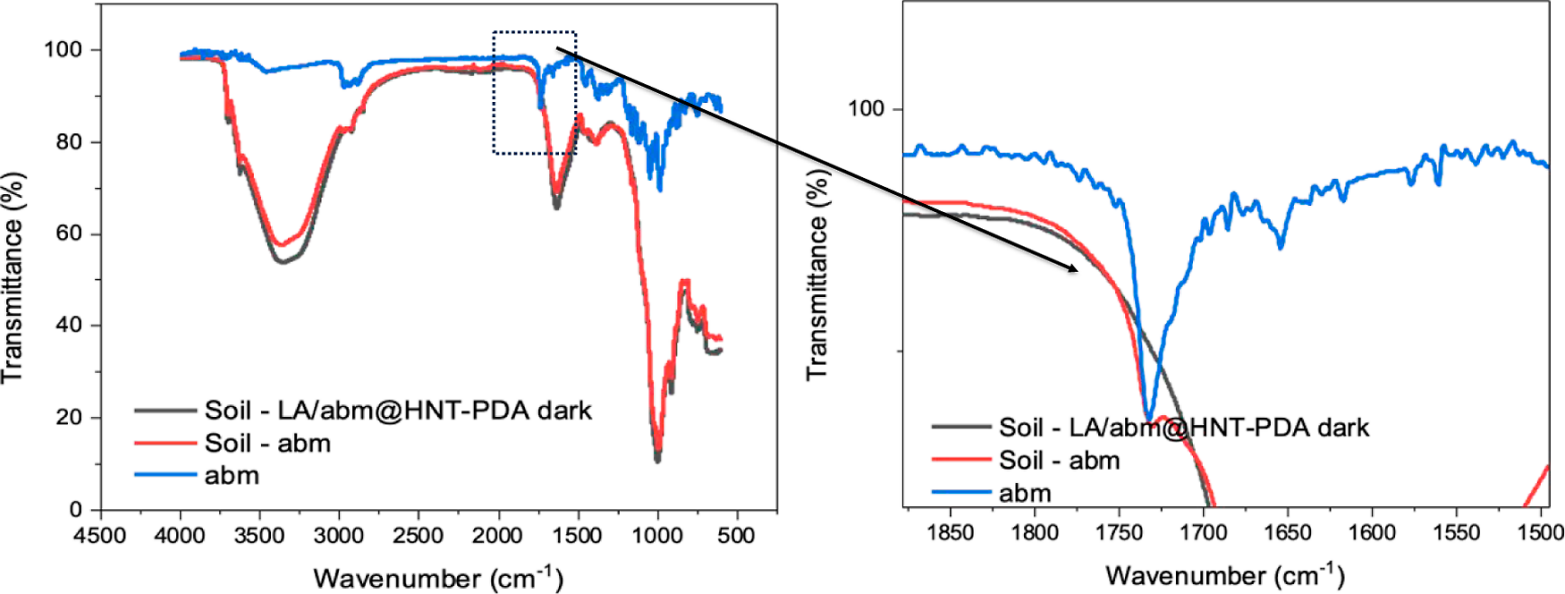
FTIR of soil samples mixed with LA/abm@HNT-PDA nanohybrids and neat abm.

To investigate the effect of the LA/abm@HNT-PDA controlled release system on environmental pollution caused by the leaching of pesticides to soil, the soil leaching of abm from the LA/abm@HNT-PDA in the dark was evaluated. The aqueous dispersion of LA/abm@HNT-PDA nanohybrids was mixed with a soil sample and incubated for 6 h followed by the removal of the liquids by vacuum filtration. The dried soil mixture was then analyzed with FTIR for the presence of abm (Figure 6). While the FTIR spectrum of the positive control sample that was prepared by mixing soil and neat abm powder presented the abm-specific peak at 1735 cm^–1^, the FTIR spectrum did not reveal any abm-related peaks when the soil sample was mixed with the LA/abm@HNT-PDA dispersion. This finding indicates that abm did not leach into the soil in the dark when encapsulated in the HNT-PDA nanocarriers. Leaching might occur only under sunlight. Therefore, the overall leaching into the soil is expected to be significantly lower than that of conventional pesticides, which continuously leach into the soil, resulting in a reduced environmental impact.

**Figure 7:**
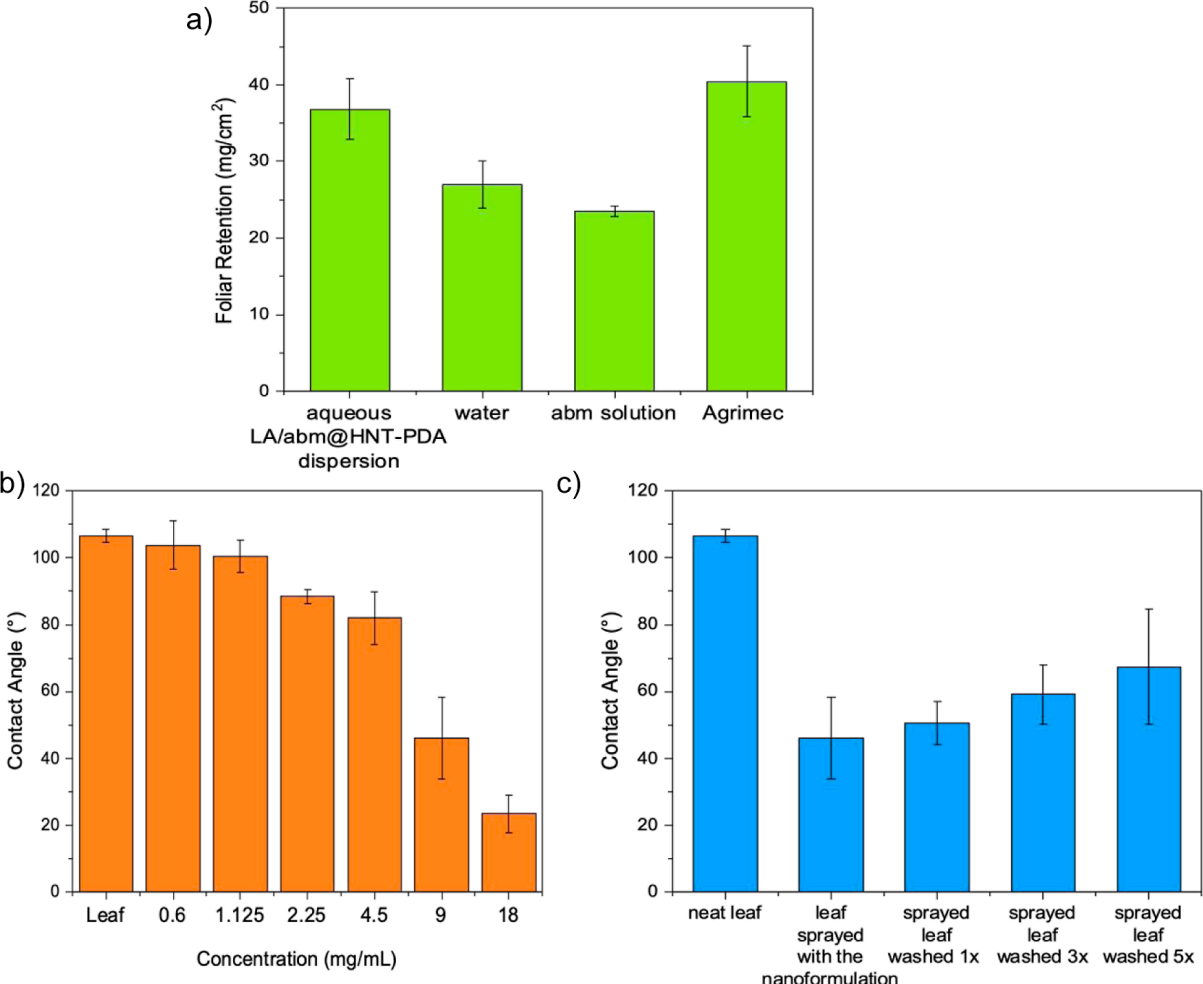
(a) Foliar retention for water, abm dissolved in methanol, and aqueous LA/abm@HNT-PDA dispersion calculated via dip-weigh method. (b) Contact angle values of eggplant leaves sprayed with aqueous dispersions of LA/abm@HNT-PDA nanohybrids at 0–18 mg/mL abm concentrations. (c) Contact angle values of eggplant leaves sprayed with aqueous LA/abm@HNT-PDA dispersions and washed with water one to five times.

The capacity of the aqueous LA/abm@HNT-PDA dispersion to adhere to plant leaves was determined by retention tests, where eggplant leaves were immersed in the dispersions followed by monitoring the weight increase of the leaves. Figure 7a shows that the aqueous LA/abm@HNT-PDA dispersion presented a retention rate higher than that of abm dissolved in methanol, demonstrating that the encapsulation in the HNT-PDA nanocarriers allowed abm to better adhere to the leaf compared to its neat form. Apparently, the highly adhesive properties of the HNT-PDA nanocarriers caused by the PDA functionalization played a role in the strong attachment and resulted in a release system with strong foliar retention properties.

The foliar adhesion characteristics of the aqueous LA/abm@HNT-PDA nanoformulations were further examined by using water contact angle measurements. Contact angle values of eggplant leaves sprayed with aqueous LA/abm@HNT-PDA dispersions at varied concentrations were determined. Figure 7b demonstrates that the water contact angle values of the leaf samples decreased as the concentration of the LA/abm@HNT-PDA dispersion sprayed on the leaf was increased, illustrating that the hydrophilic PDA layer on the HNT-PDA nanocarriers enhanced the hydrophilic nature of the nanohybrids. This finding further confirmed that the developed pesticide nanoformulation presents strong adhesion when applied to leaf surfaces, allowing efficient use in real-life conditions for agricultural purposes. To evaluate the resistance of the sunlight-triggered pesticide nanoformulations to rainwater washing, contact angle values of the eggplant leaves sprayed with the 9 mg/mL LA/abm@HNT-PDA dispersion were determined after one to five washing cycles. There was only a slight increase in the contact angle values as the number of washes of the sprayed leaves increased, which was caused by the removal of the LA/abm@HNT-PDA nanohybrids from the leaf surface (Figure 7c). However, even after five cycles of washes, the contact angle values of the sprayed leaf sample remained considerably lower than the contact angle of the neat leaf sample, indicating that a significant amount of the LA/abm@HNT-PDA nanohybrids remained attached to the leaf. This result confirmed the strong retention of the developed pesticide nanoformulation on the leaf surface and demonstrated their strong resistance to rainwater washing. The impact of the increased wettability and light scattering effect due to the presence of nanohybrids on plant health was not studied in this work. However, it is evident that a delicate balance in determining the optimum amount of nanohybrids is required to ensure sufficient insecticidal activity while also preventing potential adverse effects of increased wettability and light scattering on pathogen defense, nutrition uptake, or photosynthesis.

**Figure 8:**
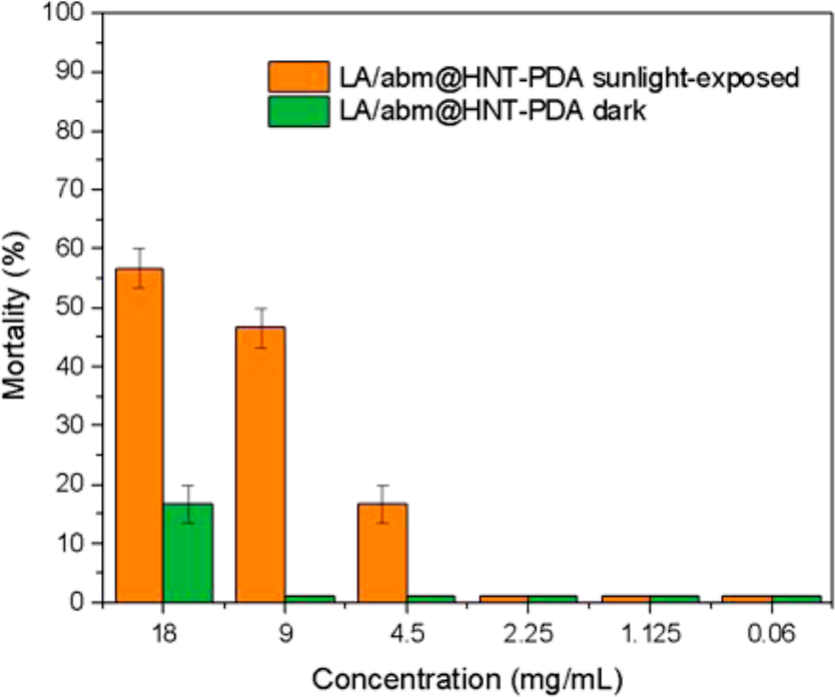
Green peach aphid mortality analysis at different concentrations of LA/abm@HNT-PDA dispersions in the dark and under sunlight irradiation.

The insecticidal activity of the LA/abm@HNT-PDA nanoformulations was determined on green peach aphids by calculating the percentage mortality. Aphids on the leaf samples sprayed with LA/abm@HNT-PDA nanohybrid dispersions at 9 and 18 mg/mL concentration exhibited a mortality rate of 50% or higher when the sprayed leaves were exposed to sunlight, whereas the dark-stored leaves sprayed with the LA/abm@HNT-PDA dispersion did not demonstrate any mortality at any concentration (Figure 8). When the aqueous dispersion of the LA/abm@HNT-PDA nanohybrids were sprayed onto the leaves, individual nanohybrids spread across the leaf surfaces presented local temperature elevations under sunlight, triggering the release of abm. The fact that only the nanohybrids absorb sunlight and convert it to heat prevented bulk heating and damage to the leaves. The 50% peach aphid mortality of the aqueous LA/abm@HNT-PDA nanoformulations at 9 mg/mL was significantly higher than the mortality of the neat abm solution and comparable to the mortality of Agrimec EC, the commercial abm formulation in cyclohexanol, under the same sunlight exposure conditions (Table S1). This result indicated that the sunlight-triggered controlled release abm nanoformulation provides significant advantages in terms of its solventless, environmentally friendly nature and sunlight-triggered, long-term effective insecticidal activity.

## Conclusions

This study introduced a sunlight-triggered release system for abm, a commonly used agrochemical in agriculture, based on its encapsulation in photothermal HNT-PDA nanocarriers and functionalization with LA as a release facilitator. The developed release system, which is composed of environmentally friendly, nontoxic components allows the release of the entrapped abm molecules when exposed to sunlight upon the light-to-heat conversion of the HNT-PDA nanocarriers, whereas the release is not triggered in the absence of the sunlight. The encapsulated abm within HNT-PDA remained stable despite the fact that abm was degraded upon exposure to sunlight irradiation, proving that nanotubes acted as ideal carriers for the prolonged preservation of abm. With the prepared release system, abm was shown to be released in a controlled manner over at least 10 days when the samples were irradiated daily with sunlight for 6 h and presented long-term killing activity on *M. persicae*. Aqueous dispersions of LA/abm@HNT-PDA nanohybrids were studied as pesticide formulations and were studied in terms of their suspensibility, foliar retention, and rainwater resistance. At a 9 mg/mL dispersion concentration, 50% green aphid mortality was observed. Presenting mortality rates comparable to those of commercial solvent-based abm formulations, the developed abm release system provides strong potential as an environmentally friendly, solventless pesticide formulation with a unique sunlight-triggered release mechanism that prevents abm from degrading in the presence of light and allows its time-dependent release.

## Supporting Information Available

SEM images of LA/abm@HNT-PDA and abm@HNT-PDA nanohybrids; green peach aphid mortality of neat abamectin; green peach aphid mortality of aqueous LA/abm@HNT-PDA dispersion, abm solution, and Agrimec at different concentrations under sunlight and in the dark; and derivation of the equations used for the calculation of the weight ratios of nanohybrid components (PDF)
