# Supplementary material for: Pesticide Nanoformulations Based on Sunlight-Activated Controlled Release of Abamectin
Source: ACS Omega. 2024 Feb 20;9(9):10380–90. doi: 10.1021/acsomega.3c08015 (PMC10918824; doi:10.1021/acsomega.3c08015)
Supplement: Supplementary file 1 — ao3c08015_si_001.pdf [file ao3c08015_si_001.pdf]

**Supplementary Information**

# Pesticide Nanoformulations based on Sunlight-Activated Controlled Release of Abamectin

*Selin Oyku Gundogdu[1,2], Ozgur Saglam[3], Ali Arda Isikber[4],Huseyin Bozkurt[4], Hayriye Unal[2]**

[1]Faculty of Engineering and Natural Sciences, Sabanci University, Istanbul, 34956, Turkey

[2]SUNUM Nanotechnology Research Center, Sabanci University, Istanbul, 34956, Turkey

[3]Namık Kemal University, Faculty of Agriculture, Tekirdağ, Turkey

[4]Kahramanmaraş Sütçü Imam University, Agriculture Faculty, Plant Protection Department, 46100 Kahramanmaraş, Turkey

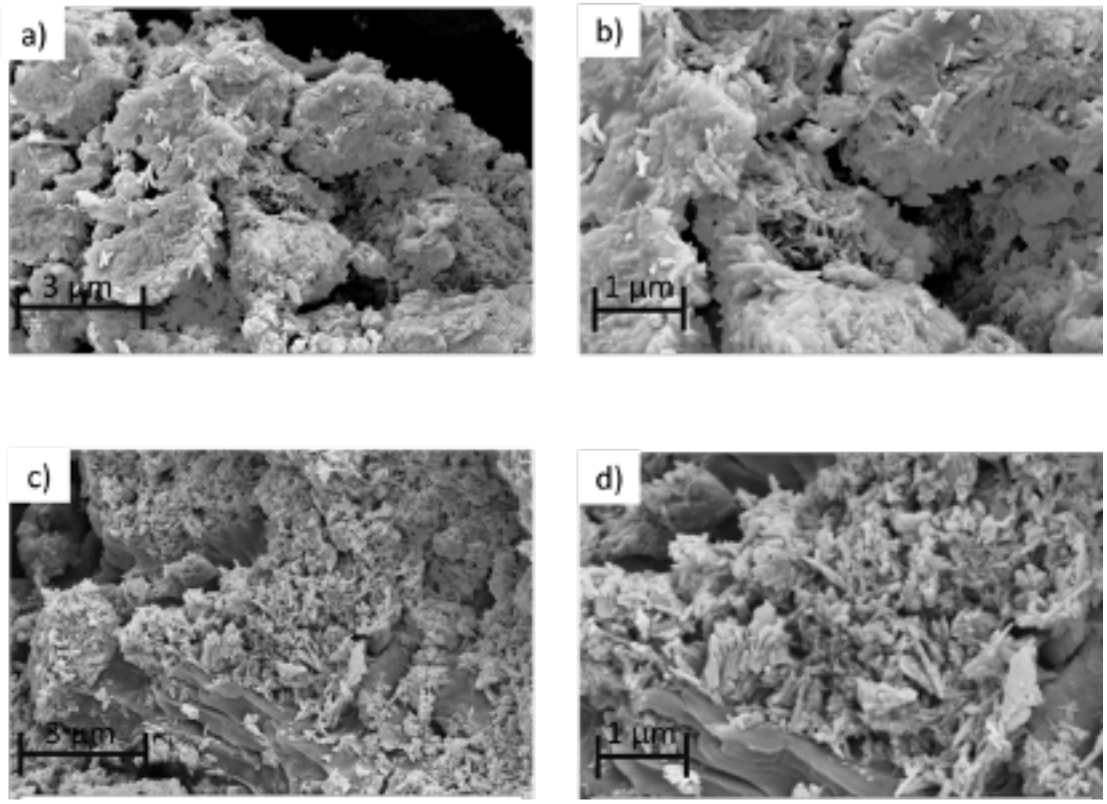


Figure S1. SEM images of LA/abm@HNT-PDA at a) 25k, b) 50k magnification and abm@HNT-PDA at c) 25k, d) 50k magnification.

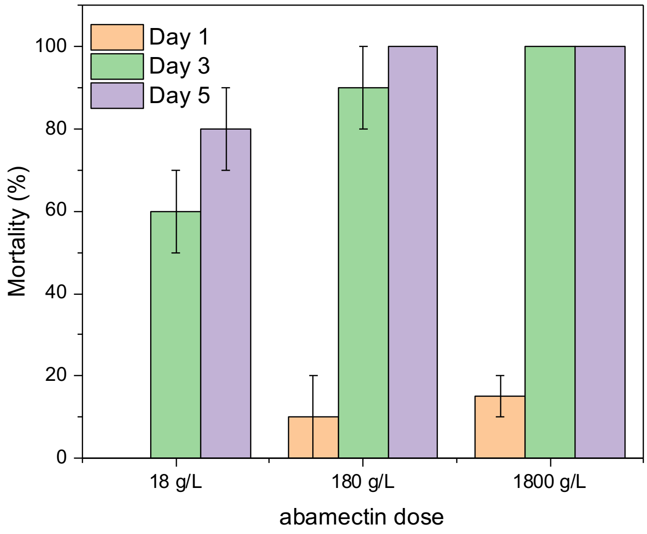


Figure S2. Peach aphid mortality for neat abm solution.

Table S1. Peach aphid mortality for aqueous LA/abm@HNT-PDA dispersion, abm dissolved in methanol, and Agrimec® EC under sunlight and in the dark.

| abm concentration in the formulation (mg/mL) | Peach aphid mortality (%) | | | | | |
|---|---|---|---|---|---|---|
| | LA/abm@HNT-PDA (sunlight-exposed) | LA/abm@HNT-PDA (dark) | abm (sunlight-exposed) | abm (dark) | Agrimec® EC (sunlight-exposed) | Agrimec ® EC (dark) |
| 18 | 60 | 20 | 0 | 70 | 90 | 80 |
| 9 | 50 | 0 | 0 | 50 | 60 | 80 |
| 4.5 | 20 | 0 | 0 | 40 | 30 | 80 |
| 2.25 | 0 | 0 | 0 | 40 | 0 | 70 |
| 1.125 | 0 | 0 | 0 | 30 | 0 | 70 |
| 0.6 | 0 | 0 | 0 | 30 | 0 | 70 |

**Supplementary Note 1**

*Calculation of %$WR_{abm}$ in LA/abm@HNT-PDA nanohybrids by TGA data:*

The weight ratio of HNT-PDA in the LA/abm@HNT-PDA nanohybrid is calculated as the ratio of remaining weights (RW) of LA/abm@HNT-PDA and HNT-PDA at 600 °C, the temperature at which the decomposition of both LA and abm is completed.

$$\%WR_{HNT-PDA} = \frac{RW_{LA/abm@HNT-PDA,\ 600\,°C}}{RW_{\mathrm{HNT-PDA},\ 600\,°C}} \text{ x } 100\% \qquad \text{Eq S1}$$

Since the LA/abm@HNT-PDA is composed of abm, LA and HNT-PDA the sum of %$WR_{abm}$ and %$WR_{LA}$ is equal to 100-%$WR_{HNT-PDA}$:

$$100\text{-}\%WR_{HNT-PDA} = \%WR_{abm} + \%WR_{LA} \qquad \text{Eq S2}$$

The %$WR_{abm}$ in LA/abm@HNT-PDA can also be calculated based on the $RW_{HNT-PDA}$, $RW_{abm}$ and $RW_{LA/abm@HNT-PDA}$ at 290 °C, the temperature at which the decomposition of LA is completed, and the decomposition of HNT-PDA did not start. The TGA of neat abm shows that at 290 °C, 44% of abm is decomposed, thus, the difference between the $RW_{HNT-PDA}$ and $RW_{LA/abm@HNT-PDA}$ at 290 °C is equal to the sum of %$WR_{LA}$ and 0.44 x %$WR_{abm}$.

$$RW_{HNT-PDA,\ 290\,°C} - RW_{LA/abm@HNT-PDA,\ 290\,°C} = \%WR_{LA} + 0.44 \text{ x } \%WR_{abm} \qquad \text{Eq S3}$$

Solving Eq S2 and Eq S3 gives the following formula for the %$WR_{abm}$ in LA/abm@HNT-PDA nanohybrids:

$$\%\boldsymbol{WR}_{\mathbf{abm}} = \left(\left(\mathbf{1} - \frac{\boldsymbol{RW}_{\mathbf{LA/abm@HNT\text{-}PDA,\ 600\,°C}}}{\boldsymbol{RW}_{\mathbf{HNT\text{-}PDA,\ 600\,°C}}}\right) \times \mathbf{100} - \boldsymbol{RW}_{\mathbf{HNT\text{-}PDA,\ 290\,°C}} + \boldsymbol{RW}_{\mathbf{LA/abm@HNT\text{-}PDA,\ 290\,°C}}\right) \times \mathbf{1.78} \qquad \text{Eq S4}$$

*Calculation of %$WR_{abm}$ in abm@HNT-PDA nanohybrids by TGA data:*

The weight ratio of HNT-PDA in the abm@HNT-PDA nanohybrid is calculated as the ratio of remaining weights (RW) of abm@HNT-PDA and HNT-PDA at 600 °C, the temperature at which the decomposition of abm is completed.

$$\%WR_{HNT-PDA} = \frac{RW_{abm@HNT-PDA,\ 600\,°C}}{RW_{\mathrm{HNT-PDA},\ 600\,°C}} \text{ x } 100\% \qquad \text{Eq S5}$$

Since the abm@HNT-PDA is composed of abm and HNT-PDA, the $\%WR_{abm}$ is equal to 100-$\%WR_{HNT\text{-}PDA}$:

$$\%WR_{abm} = 100 - \%WR_{HNT\text{-}PDA} \quad \text{Eq S6}$$
